# Efficacy and safety of diclofenac diethylamine 1.16% gel in acute neck pain: a randomized, double-blind, placebo-controlled study

**DOI:** 10.1186/1471-2474-14-250

**Published:** 2013-08-21

**Authors:** Hans-Georg Predel, Bruno Giannetti, Helmut Pabst, Axel Schaefer, Agnes M Hug, Ian Burnett

**Affiliations:** 1 Deutsche Sporthoschschule Köln, Köln, Germany; 2 CRM clinical trials GmbH, Rheinbach, Germany; 3 General Practitioner, Sports Medicine Specialist, Gilching, Germany; 4 Practitioner for Internal Medicine and Sport, Essen, Germany; 5 Novartis Consumer Health GmbH, Munich, Germany; 6 Novartis Consumer Health SA, Nyon, Switzerland

**Keywords:** Acute neck pain, Diclofenac diethylamine gel, Neck function, Pain relief, Safety

## Abstract

**Background:**

Neck pain (NP) is a common musculoskeletal disorder in primary care that frequently causes discomfort. Non-steroidal anti-inflammatory drugs (NSAIDs) may be used to reduce neck pain and associated inflammation and facilitate earlier recovery. Topical diclofenac diethylamine (DDEA) 1.16% gel is clinically proven to be effective and well tolerated in acute and chronic musculoskeletal conditions, but until now no clinical data existed for its use in acute NP. The aim of this study was to assess the efficacy and safety of DDEA 1.16% gel compared with placebo gel in acute NP.

**Methods:**

In a randomized, double-blind, placebo-controlled study, patients with acute NP (n = 72) were treated with DDEA 1.16% gel (2 g, 4x/day, for 5 days) or placebo. Efficacy assessments included pain-on-movement (POM), pain-at-rest (PAR), functional neck disability index (NDI) and response to treatment (decrease in POM by 50% after 48 h). Adverse events (AEs) were recorded throughout the study.

**Results:**

The primary outcome, POM at 48 h, was statistically significantly lower with DDEA gel (19.5 mm) vs. placebo (56.9 mm) (p < 0.0001), representing a clinically relevant decrease from baseline (75% vs. 23%, respectively). All POM scores were significantly lower with DDEA gel vs. placebo from 1 h, as were PAR and NDI scores from first assessment (24 h) onwards (all p < 0.0001). Response to treatment was significantly higher with DDEA gel (94.4%) vs. placebo (8.3%) (p < 0.0001). There were no AEs with DDEA gel.

**Conclusions:**

DDEA 1.16% gel, which is available over-the-counter, was effective and well tolerated in the treatment of acute neck pain. The tools used to assess efficacy suggest that it quickly reduced neck pain and improved neck function. However, questions remain regarding the comparability and validity of such tools. Further studies will help ascertain whether DDEA 1.16% gel offers an alternative treatment option in this common, often debilitating condition.

**Trial registration:**

ClinicalTrials.gov identifier: NCT01335724

## Background

Neck pain (NP), specifically uncomplicated neck pain (i.e. absence of fracture, no concurrent shoulder pain or nerve root symptoms, etc.), is a common, mostly musculoskeletal disorder in primary care [1]. Estimates of annual prevalence in adults range between 30–50%, with some reports indicating a prevalence as high as 71% [2,3]. It appears to affect women more than men, and prevalence increases with age [1]. Neck pain is a frequent cause of discomfort and functional impairment, and can thus limit social activities and the ability to work [4]; in fact, NP causes health-related absence from work at levels comparable to low back pain [5,6], resulting in considerable healthcare costs [7,8].

Characteristically, acute neck pain is felt as stiffness and/or pain in the cervical region of the spine and is usually associated with some of the classical symptoms of inflammation (e.g. redness, tenderness, warmth, stiffness) [9]. Acute neck pain most commonly results from injury to the muscle, disk, nerve, ligament or facet joints with subsequent inflammation and spasm [9,10]. However, no data exist on the actual cause of common, uncomplicated neck pain [10]. In acute neck pain, damage to the neck structures as a result of injury, for example, releases biochemical mediators of inflammation such as prostaglandins [9], which stimulate the nociceptors and mediate pain and inflammation [11]. One of the goals of treating any acute pain syndrome should be inhibition or suppression of production of these inflammatory mediators, and a successful outcome is one that results in less inflammation and thus less pain [9].

Although neck pain is usually self-limiting, resolving within days or weeks, it may become chronic in approximately 10% of patients [12], with symptoms persisting for more than 12 weeks. Thus, any intervention should not only aim to aid the recovery from an acute episode (usually within 4 weeks), but also to help prevent the development of long-term symptoms while minimizing the potential for adverse reactions to treatment [12].

Non-steroidal anti-inflammatory drugs (NSAIDs) are commonly used to reduce neck pain and inflammation [12]. However, to date only four trials have evaluated the efficacy and safety of oral NSAIDs as part of conservative management of acute NP [13-16], demonstrating unclear benefits [17]. None has assessed topical NSAIDs in this condition. Systematic review of the literature confirms that NSAIDs are effective analgesics and reduce inflammation in other musculoskeletal conditions [18-22].

Topical diclofenac diethylamine (DDEA) 1.16% gel is an NSAID that is clinically proven to be effective and well tolerated in acute and chronic musculoskeletal conditions [11]. Applied topically, diclofenac penetrates the skin barrier to reach joints, muscles and synovial fluid. It preferentially distributes and persists in the target inflamed tissues [23], achieving a sufficiently high concentration to exert local therapeutic activity [24]. Therefore, this randomized, double-blind, placebo-controlled study aimed to assess its efficacy and safety in the treatment of acute NP. The primary objective of the study was to assess the efficacy of DDEA 1.16% gel compared with placebo gel in the treatment of acute NP in terms of pain-on-movement (POM) at 48 h (Day 3) from baseline. Secondary objectives included the effect of DDEA 1.16% gel on pain-at-rest (PAR), functional impairment (using the neck disability index, NDI), patients’ global assessment of treatment efficacy and safety following application for 5 days.

## Methods

The clinical study (ClinicalTrials.gov identifier: NCT01335724) was designed, implemented and reported in accordance with the International Conference on Harmonization (ICH) Harmonized Tripartite Guidelines for Good Clinical Practice (GCP) and with the ethical principles laid down in the Declaration of Helsinki. The study protocol (EudraCT number 2010-022794-34), protocol amendment and associated documents were reviewed by an Independent Ethics Committee in Germany (Ethik-Kommission der Ärztekammer Nordrhein, Düsseldorf, Germany, reference number 2010456). Informed consent was obtained from each subject in writing before randomization and the rights of subjects were protected.

### Subjects

The study population consisted of male and female subjects, aged 18 years and over with acute NP originating from cervical joints and accompanying soft tissues. Pain had been present for at least 12 h and resulted in POM ≥50 mm on a 100-mm visual analogue scale (VAS). Exclusion criteria included any neck pain that was attributable to an organic disease (e.g. prolapsed disc, inflammatory arthritis, neurological diseases, etc.), as assessed by the medical history, including previous and concomitant diseases, and a neck examination and diagnosis, any recent strains of the neck muscles, chronic neck pain defined as pain for 3 months or longer, and use of pain medication within the 6 h preceding randomization.

### Study design

Eligible subjects with acute NP from three German (Cologne, Gilching and Essen) sports medicine practice clinics were randomized in a 1:1 ratio to treatment with DDEA 1.16% gel or placebo gel in a double-blind, placebo-controlled, multicenter, parallel group study. Novartis Pharmaceuticals Drug Supply Management produced the randomization list, using a system that automated the random assignment of treatment groups to randomization numbers. At Visit 1 (baseline), each subject underwent clinical evaluation and anamnesis, including previous and concomitant diseases, neck examination and diagnosis to ensure that neck pain could not be attributed to an organic disease. If all of the inclusion and none of the exclusion criteria were fulfilled, the subject was given the lowest available number on the randomization list by the investigator. Subjects, investigator staff, persons performing all assessments, monitors and data analysts remained blinded to the identity of the treatment from the time of randomization until database lock, using the following methods: (1) randomization data were kept strictly confidential until the time of unblinding and were not accessible by anyone involved in the study, (2) the identity of the treatments was concealed by the use of study drugs that were all identical in packaging, labeling, schedule of administration, appearance and odor. Unblinding only occurred in the case of subject emergencies and at the conclusion of the study.

Subjects applied the study medication for 5 days. Study visits occurred as follows: Day 1 (baseline and 1 h after first application of study drug); Day 2 (24 h ± 4 h after first application of study drug), Day 3 (48 h ± 4 h after first application of study drug) and Day 5 (study end, 96 h + 24 h after first application of study drug). Efficacy was assessed at each visit. Additional examinations included measurement of vital signs (blood pressure, pulse) by the investigator on Days 1 and 5. Adverse events (AEs) were recorded at every visit.

### Treatment regimen

Subjects were randomized to topical treatment with DDEA 1.16% gel (Voltaren® Schmerzgel® (German trade name), Voltaren® Emulgel® (European trade name), Novartis Consumer Health) or placebo gel (the same vehicle as for DDEA 1.16% gel without diclofenac). A dose of 2 g gel was applied topically with the fingertips on the affected area and massaged into the skin for around 1 min. The gel was applied 4 times a day for 5 days. Rescue medication (paracetamol, up to 2,000 mg daily) was allowed during the study, except for 6 h prior to each study visit. Non-analgesic topical treatments could be applied to other parts of the body but no other concomitant therapies were allowed.

### Study assessments

POM was assessed at all visits using a 100-mm VAS (0, no pain to 100, extreme pain) for each of three muscles (upper M. trapezius, upper M. erector spinae, M. levator scapulae); the subject stretched each muscle through a specific movement of the head, shoulders or arms as instructed by the investigators and the extent of pain was evaluated (Table 1). POM was then defined as the average of the three scores. Pain-at-rest was measured at baseline and Days 2, 3 and 5 using a 100-mm VAS (0, no pain to 100, extreme pain); the subject stood in upright position for one minute relatively motionless and the extent of pain was recorded. Functional impairment was evaluated at baseline and Days 2, 3 and 5, using the NDI [25,26] consisting of a series of questions each relating to an aspect of neck pain; the NDI score was the sum of scores for all answers given by the subject, ranging from 0 (best outcome) to 50 (worst outcome). The assessments were conducted in the following sequence: PAR, followed by POM and finally the NDI. The global assessment of treatment efficacy was recorded at Days 3 and 5 as the response to the question, “Considering how your neck pain has affected you, how would you assess the effect of treatment today?” and rated on a scale from 0 = poor to 4 = excellent. The investigator was present at all assessments, but actively participated in the POM measurement only.

**Table 1:** Measurement of pain-on-movement and pain-at-rest

	
Pain-on-movement	*M. trapezius* (upper part):
	Position: the subject sat on a chair and the investigator stood behind him/her and fixed his/her shoulders. The investigator determined which shoulder the subject pulled his/her head towards.

	Test: The subject pulled her/his head sideways towards the left or the right shoulder as appropriate without lifting up the shoulder at the same time.

	*M. erector spinae* (upper part):
	Position: The subject sat on a chair and the investigator stood behind him/her and fixed his/her shoulders with his hands. The subject’s back leaned against the back of the chair.
	Test: The subject tried to place his/her chin onto the chest without lifting up the shoulders at the same time and without losing contact with the back of the chair.

	*M. levator scapulae*
	Position: The subject sat on a chair with hanging arms and the investigator stood behind and fixed his/her shoulders with his hands.
	Test: The subject tried to lift the arms over the side upwards against the gentle resistance of the investigators hands.

	After each test:
	The extent of pain was evaluated in answer to the question:
	*“How would you describe your neck pain during movement?”*
	The subject drew a perpendicular line on the 100-mm VAS scale with anchors at 0 = “No pain at all” and 100 = “Extreme pain” to reflect the pain intensity during movement.
	The results were documented directly in the CRF.
	Pain-on-movement was defined as the average of the three VAS scores measured with the three muscle tests.
Pain-at-rest	The subject stood in an upright position for one minute, relatively motionless.
	The extent of pain is evaluated in answer to the question:
	*“How would you describe your neck pain right now?”*
	The subject drew a perpendicular line on the 100-mm VAS scale with anchors at 0 = “No pain at all” and 100 = “Extreme pain” to reflect the pain intensity at rest.
	The results were documented directly in the CRF.

CRF = case report form.

The primary efficacy variable was POM after 48 h. Secondary efficacy variables included POM at all other visits, PAR and NDI score at Days 2, 3 and 5, response to treatment (decrease in POM by 50% after 48 h), early response to treatment (decrease in POM by 10 mm at 1 h), global assessment of treatment efficacy at Days 3 and 5 and use of rescue medication.

### Safety

Adverse events, their severity and relationship to study drug were recorded at every visit. Vital signs (blood pressure and pulse rate) were assessed at the beginning and end of the study.

### Statistical analysis

The planned sample size of 72 (36 per group) was based on the primary efficacy variable, POM on VAS 48 h after baseline, assuming a two-sided t-test with α = 5% and a standard deviation (SD) of 18 mm on the VAS. Then a sample size of 36 subjects per group provides 80% power to declare statistically significant efficacy if the true difference between treatments was 12 mm. With an observed SD of 18 mm, an observed difference of 8.5 mm would be just statistically significant.

Efficacy was analyzed in the intent-to-treat (ITT) population, which included all patients who received at least one dose of study drug. All statistical tests were two-sided with significance level α = 0.05. The safety population consisted of all subjects that received at least one dose of study drug.

Efficacy was tested with an ANCOVA model, with treatment and study site as main effects and the baseline value as a covariate. As a sensitivity analysis of the terms in the final model, the treatment by study site interaction was added to the model. POM at other visits, PAR and the NDI were analyzed with the ANCOVA model used to analyze the primary efficacy outcome. The global assessment of treatment efficacy was tested with the Cochran-Mantel-Haenszel (CMH) test of mean ridits stratified by site. The percentages of subjects responding to treatment were compared with the CMH test of general association stratified by site. Because little rescue medication was used, rescue medication consumption was summarized only as whether any rescue medication was taken, overall and by day to treat neck pain, with no statistical testing. There were no missing efficacy data. Hence, procedures to impute for missing data were not needed.

## Results

### Patient characteristics

**Figure 1:**
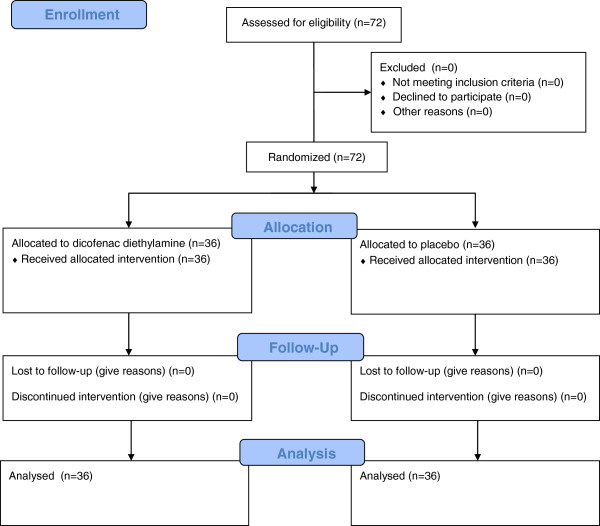
Progress of patients throughout the trial.

In total, 72 subjects were randomized to treatment between April 2011 (first subject in) and July 2011 (last subject out); 36 to DDEA 1.16% gel and 36 to placebo gel (Figure 1). All 72 patients completed the study and were included in the ITT population. The mean age was 33.8 years, with an almost equal number of males and females, and all subjects were Caucasian. Overall, subjects had neck pain at baseline for about 10 days, an NDI score (24.5) midway the best and worst outcomes (0, 50), mild-to-moderate PAR (40.6 mm) and severe POM (75.5 mm). The treatment groups did not differ appreciably on any demographic or baseline neck pain characteristics (Table 2). In particular, POM in the DDEA 1.16% gel and placebo groups were comparable at baseline (77.2 mm vs. 73.8 mm, respectively).

**Table 2:** Demographic summary by treatment group (all randomized subjects)

	**DDEA 1.16% gel**	**Placebo**
	**(N = 36)**	**(N = 36)**
Age (years) – mean ± SD (range)	29.8 ± 10.5 (19–65)	37.8 ± 15.2 (18–74)
Sex (male) – n (%)	19 (52.8)	14 (38.9)
Time from onset of neck pain (hours) – mean ± SD (range)	246.5 ± 306.5 (23–1491)	248.4 ± 348.3 (22–1691)
Pain-on-movement* (mm) (0 = no pain, 100 = extreme pain) – mean ± SD (range)	77.2 ± 10.5 (57–92)	73.8 ± 9.8 (56–90)
Neck Disability Index score (0 = best outcome, 50 = worst outcome) – mean ± SD (range)	25.3 ± 8.4 (7–37)	23.6 ± 7.3 (9–34)
Pain-at-rest (mm) (0 = no pain, 100 = extreme pain) – mean ± SD (range)	41.1 ± 17.0 (11–79)	40.1 ± 16.9 (5–64)

* Mean score, average of the three VAS scores. *DDEA* = diclofenac diethylamine; *SD* = standard deviation.

### Efficacy

The primary outcome, POM at 48 hours, was almost three times lower with DDEA 1.16% gel (19.5 mm) compared with placebo (56.9 mm) (p < 0.0001, Table 3). Change from baseline was 57.7 mm with DDEA 1.16% gel (75% decrease) compared with 16.9 mm with placebo (23% decrease).

**Table 3:** Pain-on-movement at 48 h (primary efficacy variable)

**Mean (SD) [mm]**		LS * mean (SE) [mm]		**LS mean difference (SE) 95% CI**	**p-value **^**†**^
DDEA 1.16% gel (N = 36)	Placebo	DDEA 1.16% gel (N = 36)	Placebo	Placebo – DDEA 1.16% gel	
	(N = 36)		(N = 36)		
19.5 (12.9)	56.9 (16.1)	17.8 (2.1)	56.3 (2.4)	38.4 (3.0)	p < 0.0001
				32.4–44.5	

* LS-mean – Least squares mean difference adjusted for effects of site and baseline POM; ^†^*ANCOVA* – Analysis-of-covariance test with treatment and site as main effects and the baseline POM as a covariate.

*DDEA* = diclofenac diethylamine; *SD* = standard deviation; *SE* = standard error; *CI* = confidence interval.

**Figure 2:**
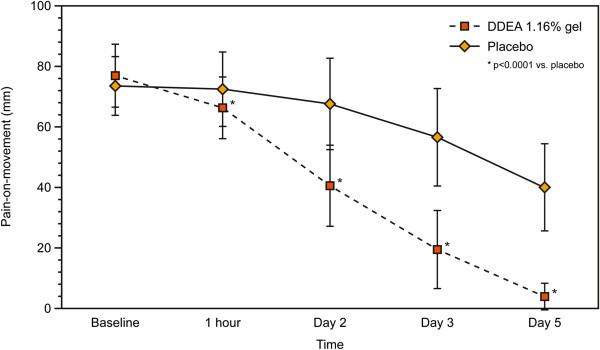
**Pain-on-movement (POM) over time (5 days of treatment).** There was a significantly greater reduction in POM with DDEA 1.16% gel compared with placebo from the first assessment at 1 h to the final visit at 96 h (p < 0.0001).

**Figure 3:**
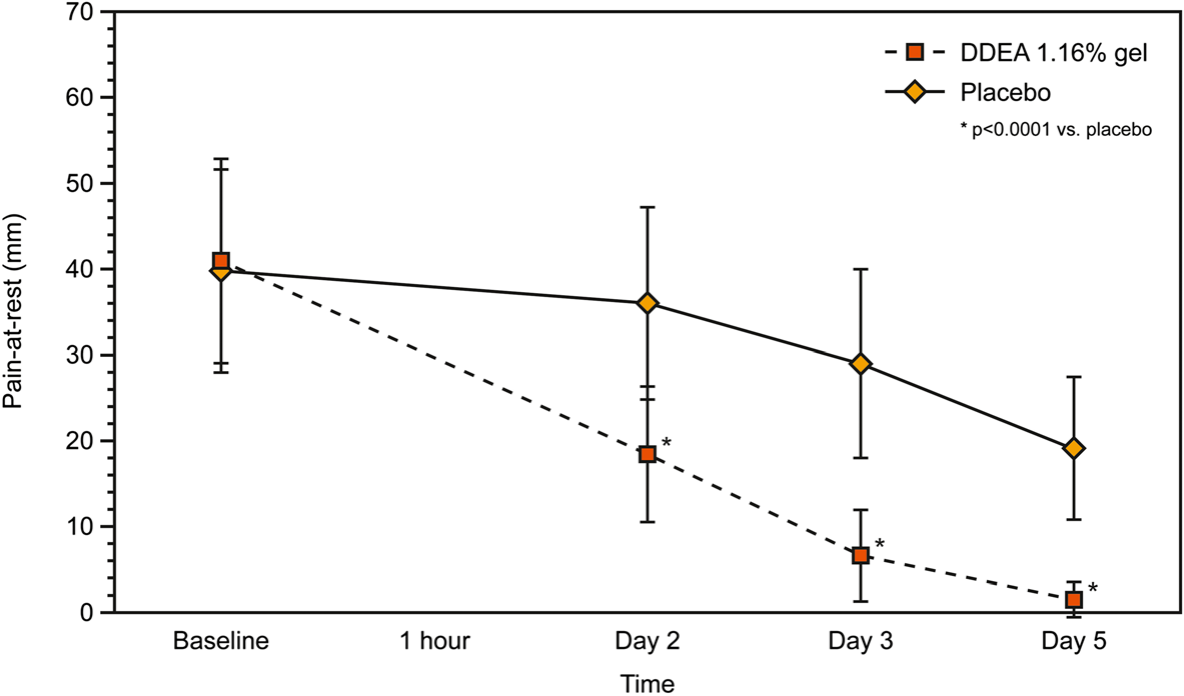
**Pain-at-rest (PAR) over time (5 days of treatment).** PAR was significantly lower with DDEA 1.16% gel vs. placebo at all post-baseline visits (p < 0.0001).

**Figure 4:**
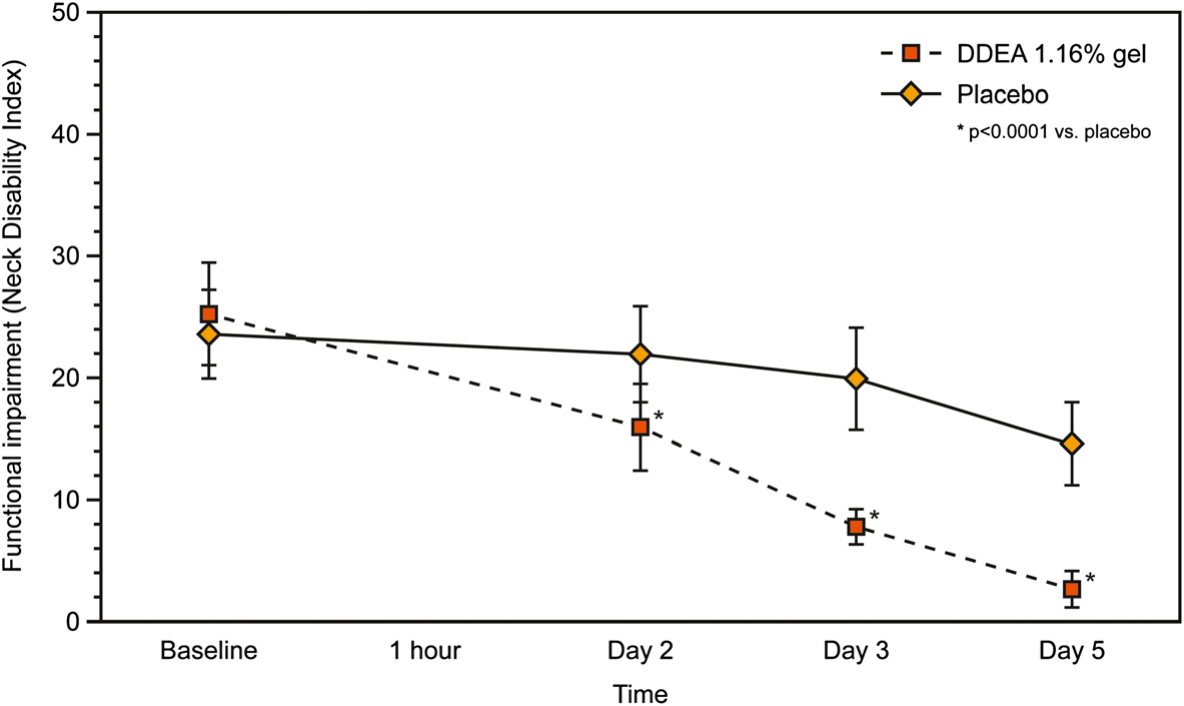
**Functional impairment (Neck Disability Index, NDI) over time (5 days of treatment).** The NDI score demonstrated that patients’ function improved significantly with DDEA 1.16% gel vs. placebo from the first to the last assessment (p < 0.0001).

With regards to the secondary efficacy variables, there was a significantly greater reduction in POM with DDEA 1.16% gel compared with placebo from the first assessment at 1 h to the final visit at 96 h (p < 0.0001) (Figure 2). Similarly, PAR was significantly lower with DDEA 1.16% gel vs. placebo at all post-baseline visits (p < 0.0001) (Table 4, Figure 3). The NDI score demonstrated that patients’ function also improved significantly with DDEA 1.16% gel vs. placebo from the first to the last assessment (p < 0.0001) (Table 4, Figure 4).

**Table 4:** Changes in secondary efficacy variables

	**Time after first application of study drug**					
	**24 hours**		**48 hours**		**96 hours**	
**Efficacy variable**	**DDEA 1.16% gel**	**Placebo**	**DDEA 1.16% gel**	**Placebo**	**DDEA 1.16% gel**	**Placebo**
Pain-at-rest – mean ± SD, mm (% difference from baseline)	18.5 ± 11.3* (55.0)	36.3 ± 16.0 (9.5)	6.5 ± 7.6* (84.2)	29.2 ± 15.7 (27.2)	1.2 ± 2.9* (97.1)	19.2 ± 11.9 (52.1)
Neck disability index score – mean ± SD (% difference from baseline)	16.0 ± 7.1* (36.8)	21.9 ± 7.9 (7.2)	7.8 ± 2.9* (69.2)	19.9 ± 8.4 (15.7)	2.8 ± 3.0* (88.9)	14.6 ± 6.8 (38.1)

* *ANCOVA* (analysis-of-covariance test with treatment and site as main effects and the baseline pain-at-rest value as a covariate): p < 0.0001 versus placebo. *SD* = standard deviation.

Almost all DDEA 1.16% gel patients (94.4%) responded to treatment (decrease in POM by 50% after 48 h) compared with 8.3% of placebo patients (p < 0.0001). More than half of DDEA 1.16% gel patients (58.3%) showed an early response to treatment (decrease in POM by 10 mm at 1 h) compared with placebo (8.3%), with a mean reduction from 77.2 ± 10.4 to 66.7 ± 10.2 with DDEA 1.16% gel vs. 73.8 ± 9.7 to 72.6 ± 12.3 with placebo (p < 0.0001). At study end, 100% of DDEA 1.16% gel patients rated treatment as “good” to “excellent” compared with just 19.4% of placebo patients (p < 0.0001). Only one patient used rescue medication once (in the placebo group, for ankle pain and headache). Efficacy outcomes were consistent across all study sites.

### Safety

Only one AE was recorded (moderate headache in the placebo group), which was not considered by the investigator to be drug related. No serious AEs were reported and no clinically relevant abnormal vital signs were observed during the study, according to the clinical judgment of the investigators.

## Discussion

The present study is the first randomized, controlled trial to demonstrate the efficacy of a topical NSAID (DDEA 1.16% gel) in relieving acute NP and helping to improve neck function. Furthermore, it demonstrates that the onset of effect of DDEA 1.16% gel is relatively fast, for a topical agent, a feature that is desirable for the analgesics aiming to treat acute pain [27]. Within 1 hour of the first application, a response to treatment (i.e. a decrease in POM by 10 mm [28,29]) was detected in more than half of patients; however, the clinical relevance of these findings should be carefully considered given the limited number of patients included in the study and the possible different etiologies of neck pain. The results are nevertheless encouraging and a good basis for further investigation. Measurement of POM at 48 h (the primary efficacy variable) showed that the baseline POM was reduced by 75% with DDEA 1.16% gel vs. 23% with placebo. This reduction can reasonably be considered clinically relevant. By the end of the study, after 4–5 days of treatment, almost complete pain relief was achieved with DDEA 1.16% gel (as assessed by POM and PAR) and almost all patients responded to treatment (94%, compared with only 8% of placebo patients).

The detected improvement in neck function with DDEA 1.16% gel was approximately 100% higher than placebo. Furthermore, patients randomized to DDEA 1.16% gel experienced an approximate 50% reduction in recovery time (defined as a clinically relevant decrease in pain plus an increase in neck function) compared with placebo patients. The mean POM and neck disability scores were achieved at 48 h with DDEA 1.16% gel compared with approximately 96 h with placebo.

No adverse effects were experienced with active treatment. The efficacy and tolerability of DDEA 1.16% gel led to all patients rating treatment as “good” to “excellent”, whereas only one-fifth of placebo patients rated treatment similarly; it should be noted that this reflects the satisfaction of the patients, rather than a difference in safety between the two treatment groups.

The fact that treatment did not cause any adverse effects in this study is an important finding confirming the good tolerability of DDEA 1.16% gel, which is available over-the-counter. Thus, DDEA 1.16% gel may be a preferable alternative to oral NSAIDs which, although generally well tolerated, can cause gastrointestinal adverse reactions such as bleeding and perforation [30]. The results of this study in acute NP could be used as hypothesis to investigate the effects of the treatment in other pain models (e.g. non-specific lower back pain).

The early response to treatment noted in this study cannot be compared to the literature, in which the earliest assessment of neck pain or disability was 1 week [14,31,32]. In fact, the short follow-up period in this study was a limitation, as it was not long enough to determine the time to resolution in the placebo group. Although extrapolation of the data suggests that resolution would have been achieved in approximately a week with placebo, this can only be confirmed with a longer follow-up period. Other publications have followed patients for up to 52 weeks [14,31,32], allowing a better reflection of the efficacy and safety of the active treatment and placebo over time. In future studies, inclusion of a non-treated study arm would help to further clarify the efficacy of DDEA 1.16% gel. Since the study took an assessment approach based on parameters related to inflammation, and since psychological factors may play an important role in neck pain, another important limitation of the study is the lack of validation of the psychometric properties of the measured parameters (for example, the standard error of measurement, the smallest detectable change, etc.). This situation should be addressed in further studies.

As mentioned previously, another limitation of the study is the relatively small size of the study population. Since the primary efficacy variable was the most important, the power calculation was based primarily on this outcome; therefore, conclusions based on secondary outcomes should be made with this methodological factor in mind. It should also be noted that the studies used to determine a statistically significant early response to treatment (decrease in POM by 10 mm at 1 h) [28,29] were performed in trauma patients; nevertheless, we believe that it is a suitable parameter to use since the pathophysiological mechanisms of inflammation underlying both conditions can be assumed to be similar. As a challenge to the active treatment, patients showing relatively strong pain symptoms (POM ≥ 50 mm) were included – this does not necessarily mean that similar results will be achieved under different conditions. Therefore, in further studies the efficacy of topical DDEA 1.16% in treating patients with mild neck pain should also be investigated, in such cases where a pharmacological treatment is considered appropriate.

Based on these limitations, it would be desirable to confirm our observations around fast relief of pain with DDEA 1.16% gel in a study with a larger cohort, perhaps with less severe neck pain, and with suitable evaluation of the psychometric properties of the outcome measurements.

In summary, DDEA 1.16% gel patients with acute NP experienced a statistically significant reduction in pain from the first dose of treatment (at 1 h), were virtually pain free with little functional impairment in the neck 96 h after initiating therapy, and all patients were satisfied with treatment. In contrast, subjects treated with placebo still had considerable pain and functional impairment at the end of the study.

## Conclusions

The findings of this study suggest that topical DDEA 1.16% gel is fast-acting, effective and well tolerated for the relief of acute neck pain, helping to improve neck function. This study demonstrated for the first time the significant superiority and clinical relevance of treatment with DDEA 1.16% gel vs. placebo on all parameters assessed. Thus DDEA 1.16% gel can be considered a potential treatment for acute neck pain, and could be investigated for use in other indications with similar pathomechanisms, such as non-specific lower back pain.

## Abbreviations

AE: Adverse event; CMH: Cochran-Mantel-Haenszel; DDEA: Diclofenac diethylamine; ITT: Intention-to-treat; NDI: Neck disability index; NP: Neck pain; NSAID: Non-steroidal anti-inflammatory drug; PAR: Pain-at-rest; POM: Pain-on-movement; SD: Standard deviation; VAS: Visual analogue scale.

## Competing interests

The authors declare that they have no competing interests.

## Authors’ contributions

H-GP: principal investigator, patient recruitment, data collection. BG: data collection, quality assurance of data, final study report. HP & AS: co-investigators, patient recruitment data collection. AMH: investigator training. IB: study design, analysis and interpretation of data. All authors discussed the results and critically reviewed the manuscript. All authors read and approved the final manuscript.

## Pre-publication history

The pre-publication history for this paper can be accessed here:

http://www.biomedcentral.com/1471-2474/14/250/prepub

## References

[B1] Manchikanti L Singh V Datta S Cohen S Hirsch J Comprehensive review of epidemiology, scope and impact of spinal pain Pain Physician 2009 12 E35 E70 19668291

[B2] Fejer R Kyvik K Hartvigsen J The prevalence of neck pain in the world population: a systematic critical review of the literature Eur Spine J 2006 15 834 848 10.1007/s00586-004-0864-4 15999284 PMC3489448

[B3] Hogg-Johnson S van der Velde G Carroll L Holm L Cassidy J Guzman J Cote P Halderman S Ammendolia C Carragee E The burden and determinants of neck pain in the general population: results of the bone and joint decade 2000–2010 task force on neck pain and its associated disorders J Manipulative Physiol Ther 2009 32 S46 S60 10.1016/j.jmpt.2008.11.010 19251074

[B4] Chiu T Leung A Neck pain in Hong Kong: A telephone survey on prevalence, consequences, and risk groups Spine 2006 31 E540 E544 10.1097/01.brs.0000225999.02326.ad 16845340

[B5] Kvarnstrom S Occurrence of musculoskeletal disorders in a manufacturing industry with special attention to occupational shoulder disorders Scand J Rehabil Med Suppl 1983 8 1 114 6579645

[B6] Ihlebaek C Brage S Eriksen H Health complaints and sickness absence in Norway, 1996–2003 Occup Med (Lond) 2007 51 43 49 17046991 10.1093/occmed/kql107

[B7] Ferrari R Russell A Regional musculoskeletal conditions: neck pain Best Pract Res Clin Rheumatol 2003 17 57 70 10.1016/S1521-6942(02)00097-9 12659821

[B8] Hansson E Hansson T The costs for persons sick-listed more than one month because of low back or neck problems. A two-year prospective study of Swedish patients Eur Spine J 2005 14 337 345 10.1007/s00586-004-0731-3 15150703 PMC3489204

[B9] Omoigui S The biochemical origin of pain: The origin of all pain is inflammation and the inflammatory response. PART 2 of 3 - Inflammatory profile of pain syndromes Med Hypotheses 2007 69 1169 1178 10.1016/j.mehy.2007.06.033 17728071 PMC2771434

[B10] Bogduk N The anatomy and pathophysiology of neck pain Phys Med Rehabil Clin N Am 2003 14 455 472 10.1016/S1047-9651(03)00041-X 12948338

[B11] Zacher J Altman R Bellamy N Bruhlmann P Da Silva J Huskisson E Taylor R Topical diclofenac and its role in pain and inflammation: an evidence-based review Curr Med Res Opinion 2008 24 925 950 10.1185/030079908X273066 18279583

[B12] Binder A Neck pain Clin Evid (Online) 2008 pii 110319445809

[B13] Dostal C Pavelka K Lewit K Ibuprofen in the treatment of the cervicocranial syndrome in combination with manipulative therapy Fysiatr Revmatol Vestn 1978 56 258 263 359444

[B14] Bronfort G Evans R Anderson A Svendson K Bracha Y Grimm R Spinal manipulation, medication, or home exercise with advice for acute and subacute neck pain: a randomized trial Ann Intern Med 2012 156 1 10 10.7326/0003-4819-156-1-201201030-00002 22213489

[B15] Urbin Choffray D Crielaard J Albert A Franchimont P Comparative study of high bio-availability glaphenine and paracetamol in cervical and lumbar arthrosis Clin Rheumatol 1987 6 518 525 10.1007/BF02330588 2896557

[B16] San Martin J Roldan A Comparison of eterilate and acetylsalicylic acid in the treatment of cervicoarthrosis: double blind test Arch Farmacol Toxicol 1978 4 41 46 358926

[B17] Peloso P Gross A Haines T Trinh K Goldsmith C Burnie S Cervical Overview Group Medicinal and injection therapies for mechanical neck disorders Cochrane Database Syst Rev 2007 3 Art. No.: CD000319. DOI: 10.1002/14651858.CD000319.pub4 10.1002/14651858.CD000319.pub4 17636629

[B18] Bjordal J Ljunggren A Klovning A Slørdal L Non-steroidal anti-inflammatory drugs, including cyclo-oxygenase-2 inhibitors, in osteoarthritic knee pain: meta-analysis of randomised placebo controlled trials BMJ 2004 329 1317 10.1136/bmj.38273.626655.63 15561731 PMC534841

[B19] Jordan K Arden N Doherty M Bannwarth B Bijlsma J Dieppe P Gunther K Hauselmann H Herrero-Beaumont G Kaklamanis P EULAR recommendations 2003: an evidence based approach to the management of knee osteoarthritis: report of a task force of the standing committee for international clinical studies including therapeutic trials (ESCISIT) Ann Rheum Dis 2003 62 1145 1155 10.1136/ard.2003.011742 14644851 PMC1754382

[B20] Mason L Moore R Edwards J Derry S McQuay H Topical NSAIDs for acute pain: a meta-analysis BMC Fam Pract 2004 5 10 10.1186/1471-2296-5-10 15147585 PMC420463

[B21] Moore R Tramer M Carroll D Wiffen P McQuay H Quantitative systematic review of topically applied non-steroidal anti-inflammatory drugs BMJ 1998 316 333 338 10.1136/bmj.316.7128.333 9487165 PMC2665568

[B22] van Tulder M Scholten R Koes B Deyo R Non-steroidal anti-inflammatory drugs for low-back pain Cochrane Database Syst Rev 2000 2 *Art No.: CD000396* DOI: 10.1002/14651858.CD000396 10.1002/14651858.CD000396 10796356

[B23] Brune K Persistence of NSAIDs at effect sites and rapid disappearance from side-effect compartments contributes to tolerability Curr Med Res Opin 2007 23 2985 2995 10.1185/030079907X242584 17949535

[B24] Zacher J Altman R Bellamy N Bruhlmann P Da Silva J Huskisson E Topical diclofenac and its role in pain and inflammation: an evidence-based review Curr Med Res Opin 2008 24 925 950 10.1185/030079908X273066 18279583

[B25] Vernon H Mior S The Neck Disability Index: a study of reliability and validity J Manipulative Physiol Ther 1991 14 409 415 1834753

[B26] Vernon H The Neck Disability Index: state-of-the-art, 1991–2008 J Manipulative Physiol Ther 2008 31 491 502 10.1016/j.jmpt.2008.08.006 18803999

[B27] Moore N In search of an ideal analgesic for common acute pain Acute Pain 2009 11 129 137 10.1016/j.acpain.2009.09.003

[B28] Todd K Funk K Funk J Bonacci R Clinical significance of reported changes in pain severity Ann Emerg Med 1996 27 485 489 10.1016/S0196-0644(96)70238-X 8604867

[B29] Gallagher E Liebman M Bijur P Prospective validation of clinically important changes in pain severity measured on a visual analogue scale Ann Emerg Med 2001 38 633 638 10.1067/mem.2001.118863 11719741

[B30] Hippsley-Cox J Coupland C Logan R Risk of adverse gastrointestinal outcomes in patients taking cyclo-oxygenase-2 inhibitors or conventional non-steroidal anti-inflammatory drugs: population based nested case–control analysis BMJ 2005 331 1310 10.1136/bmj.331.7528.1310 16322018 PMC1298853

[B31] Vos V Verhagen A Passchier J Koes B Clinical course and prognostic factors in acute neck pain: an inception cohort study in general practice Pain Med 2008 9 572 580 10.1111/j.1526-4637.2008.00456.x 18565009

[B32] Hush J Michaleff Z Verhagen A Refshauge K Prognosis of acute idiopathic neck pain is poor: a systematic review and meta-analysis Arch Phys Med Rehabil 2011 92 824 829 10.1016/j.apmr.2010.12.025 21458776

